# Use of miRNAs in biofluids as biomarkers in dietary and lifestyle intervention studies

**DOI:** 10.1007/s12263-015-0483-1

**Published:** 2015-08-02

**Authors:** Sophie Rome

**Affiliations:** CarMeN Laboratory (INSERM 1060, INRA 1397, INSA), Faculté de Médecine Lyon-Sud, University of Lyon, Chemin du Grand Revoyet, 69600 Oullins, France

**Keywords:** Biomarkers, Circulating microRNA, Nutrition, Gut microbiota, Lifestyle interventions, Diet

## Abstract

The selection of biomarkers in nutrigenomics needs to reflect subtle changes in homoeostasis representing the relation between nutrition and health, or nutrition and disease. It is believed that noncoding RNAs, such as circulating microRNAs (miRNAs), may represent such a new class of integrative biomarkers. Until now, the most relevant body fluids for miRNA quantification in response to nutrition have not been clearly defined, but recent studies listed in this review indicate that miRNAs from plasma or serum, PBMC and faeces might be relevant biomarkers to quantify the physiological impacts of dietary or lifestyle intervention studies. In addition, a number of recent studies also indicate that miRNAs could permit to monitor the impact of diet on gut microbiota. We also discuss the main preanalytical considerations that are important to take into account before miRNA screening which can affect the reproducibility of the data.

## Introduction

Soon after the completion of the first draft of the human genome in 2001 (Lander et al. 2001), *nutrigenomics* emerged as new area of research in nutrition science (Peregrin 2001). Researches in nutrigenomics (not to be confused with *nutrigenetics*) focus on identifying and understanding molecular-level interactions between ingested nutrients, and other dietary bioactive compounds, with the genome. In 2001, it was expected that in ‘less than 10 years’, results from nutrigenomic studies would provide a basis for the development of safe and effective diet therapies to rationally and specifically improve health in individuals or subgroups of the population (Peregrin 2001). Clearly, hope for the discovery of ‘biomarkers’ for quantifying the impact of specific nutrients on the genome for the selection of bioactive nutrients was underlying this predicted revolution. As of May 2015, 1241 papers were found in MEDLINE (*Medical Literature Analysis and Retrieval System Online*) using the term ‘nutrigenomic’, of which 40 % are reviews. Among the 1241 nutrigenomic papers, only 91 contain both terms ‘biomarker and nutrigenomics’, of which 30 % are reviews. The quest to identify biomarkers has been hindered by the concomitant development of new expensive ‘omic’ tools (e.g. microarrays, proteomic, metabolomic, sequencing), which required the development of specific, sophisticated bioinformatic software, and by our limited knowledge of the organization of the human genome. Indeed, over 98 % of the human genome is noncoding RNA (Mattick 2001) which was not analysed in the first generation of microarrays or by proteomics techniques as noncoding RNAs, although functional RNA molecules, are not supposed to be translated into proteins. Moreover, it has become clear that single nutrients have multiple known, and likely unknown, physiological actions which are not easily addressed by single-gene, protein or metabolite approaches. Therefore, a combination of all genomic-based data may be necessary to detect the full metabolic effect of dietary intervention and/or to find relevant nutritional biomarkers. Circulating microRNAs (miRNAs) may represent such a new class of integrative biomarker that reflects either the early phases of metabolic stress and nutrition interventions to restore homoeostasis, or the development of metabolic syndrome associated with complex diseases. Recent studies described below show that miRNAs might also be useful for detecting the effects of dietary or lifestyle intervention studies.

**Fig. 1:**
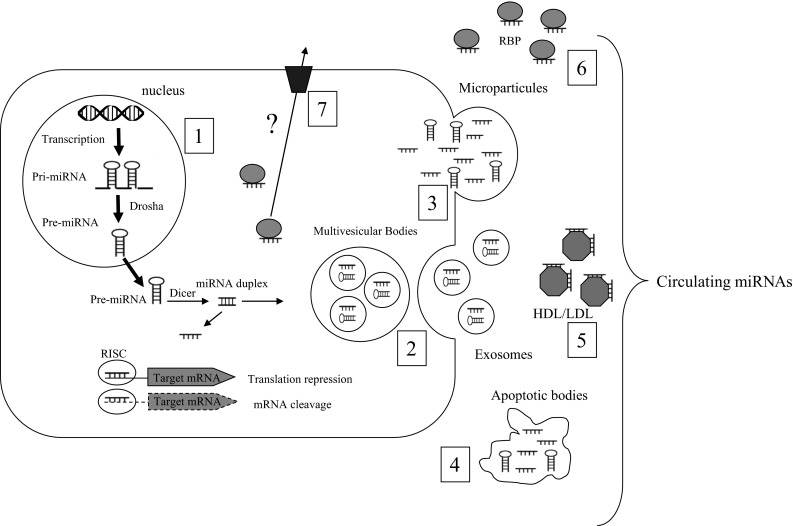
Origin of the different populations of extracellular miRNAs in biofluids. (*1*) In the nucleus, miRNA genes are transcribed by the RNA polymerase II into primary miRNAs (pri-miRNAs) from DNA and processed by the Drosha complex (pre-miRNAs). pre-miRNAs are exported to the cytoplasm and cleaved by Dicer to produce a double-stranded miRNA duplex. The duplex is separated, and a mature miRNA is incorporated into the RNA-induced silencing complex (RISC). Within the RISC complex, miRNAs bind to their target messenger RNAs (mRNAs) to repress their translation or induce their degradation. In the cytoplasm, pre-miRNAs and mature miRNAs can also be incorporated into small vesicles called exosomes, which are released from cells when multivesicular bodies (MVB) fuse with the plasma membrane (*2*). Pre-miRNA or mature miRNA can also be released through blebbing of the plasma membrane (microparticles) (*3*) or during cell apoptosis in apoptotic bodies (*4*). miRNAs are also found in circulation in vesicle-free form. These miRNAs can be associated with high-density lipoproteins (HDL) (*5*) or bound to RNA-binding proteins (RNP) (*6*). In addition, miRNAs may be released actively, in an miRNA-specific manner, through interaction with specific membrane channels or proteins (*7*)

MicroRNAs (miRNAs) are a class of evolutionally conserved noncoding RNAs of 19–22 nucleotides and function as negative regulators of gene expression. Originally discovered in *C. elegans*, miRNAs regulate fundamental cellular processes in diverse organisms (Lagos-Quintana et al. 2001; Lee and Ambros 2001). miRNAs are encoded within the genome (from intronic, exonic or intergenic regions) and are initially transcribed as primary transcripts that can be several kilobases in length (pri-miR) (Fig. 1). Primary transcripts are successively cleaved by two RNase III enzymes, Drosha in the nucleus and Dicer in the cytoplasm, to produce 70 nucleotide long precursor miRNAs (pre-miR) and then mature miRNAs, respectively. Single-stranded mature miRNAs associate with Argonaute proteins (Ago) to form the core of a multicomponent gene regulatory complex named the RNA-induced silencing complex (RISC) (Bartel 2004). Three mechanisms have been described for gene regulation via miRNA (1) translation repression, (2) direct mRNA degradation (III) and miRNA-mediated mRNA decay. Recent data have suggested that the mechanism of repression is predominately via a decrease in mRNA target stability (Guo et al. 2011). miRNA activity and abundance is also regulated on various levels ranging from transcription and processing to target site binding and miRNA stability (Treiber et al. 2012). Bioinformatic analyses indicate that miRNAs can regulate multiple target mRNAs (i.e. nearly 60 % of all mammalian mRNAs represent miRNA targets) and individual mRNA can be targeted by several miRNAs (Lewis et al. 2003). Functional analysis of miRNA target genes has shown that they play a major role in the regulation of developmental processes including cell growth and differentiation and programmed cell death by targeting preferential signalling pathways and transcription factors (Stark et al. 2005; Cui et al. 2006, 2007). In addition, important roles of miRNAs have emerged in the control of metabolic pathways involved in lipid metabolism, adipocyte differentiation, energy homoeostasis, glucose-stimulated insulin secretion and inflammation (Lynn 2009). Thus, as a consequence of the widespread range of processes they are able to influence, miRNA deregulation is a hallmark of several pathological conditions, including cancer (Lages et al. 2012), inflammation (O’Connell et al. 2011), neurological disorders (Salta and De Strooper 2012), cardiovascular diseases and metabolic disorders (Lorenzen et al. 2010). Studies during the last 5 years have also demonstrated that dietary compounds such as amino acids, carbohydrates, fatty acids and vitamins can lead to changes in miRNA expressions and affect their functions (Garcia-Segura et al. 2013), making them good candidates to study, at the tissue or cellular level, the response of a specific nutrient or diet (Garcia-Segura et al. 2013).

## Cellular miRNAs are exported outside the cells and detected in all biofluids

Recently, significant amounts of miRNAs have been found not only intracellularly, but in extracellular human body fluids (e.g. serum/plasma, saliva, urine, tears, amniotic fluids and milk) (Weber et al. 2010). They are remarkably stable despite high extracellular RNase activities. The stability of miRNAs was demonstrated using sera from various sources, after treatment under harsh conditions including boiling, low/high pH, extended storage and freeze–thaw cycles. The detection of miRNAs in serum samples treated under these harsh conditions yielded no significant differences compared to nontreated serum (Chen et al. 2008; Gilad et al. 2008; Mitchell et al. 2008). Interestingly, they are also detected in the serum/plasma in various animal species (Andersson et al. 2015; Fujiwara-Igarashi et al. 2015; Garza-Manero et al. 2015; Yamada et al. 2015). Given that a lot of animals are used for biomedical research, for drug screening and in dietary intervention studies, analysis of miRNA expression profiles of these clinically relevant species may facilitate an understanding of their relationship to health and disease and lead to their use as biomarkers in human. An attractive aspect of this possibility is that compared to protein-based biomarkers, the complexity of miRNA is much lower.

Extracellular miRNAs are enclosed in vesicles (e.g. in exosomes, shedding vesicles, apoptotic bodies), or associated with, or packaged within high-density and low-density lipoprotein or associated with RNA-binding proteins (e.g. high-density lipoprotein, Argonaute 2 and nucleophosmin 1) (Cocucci et al. 2009; Zernecke et al. 2009; Arroyo et al. 2011; Vickers et al. 2011; Wagner et al. 2013) (Fig. 1). In the latter case, extracellular miRNAs may either represent by-products of dead/dying cells which persist due to their stability within the protein complex, or suggest that cells may release miRNAs through a protein carrier pathway (Wang et al. 2010). For miRNAs exported in extracellular vesicles, compelling evidence supports their role in affecting a broad range of physiological and pathological processes. It is believed that these extracellular miRNA-associated unication (Lotvall and Valadi 2007; Valadi et al. 2007; Iguchi et al. 2010; Mathivanan et al. 2010; Vickers et al. 2011) and recent studies have shown that the level and composition of these extracellular/circulating miRNAs correlate well with diseases or injurious conditions (Mitchell et al. 2008; Laterza et al. 2009). A global survey of the miRNA distribution in 12 human body fluids (i.e. amniotic fluid, breast milk, bronchial lavage, cerebrospinal fluid, colostrum, peritoneal fluid, plasma, pleural fluid, saliva, seminal fluid, tears and urine) showed distinct compositions in different fluid types (Weber et al. 2010). Notably, the miRNA spectrum in plasma is different from that of most of the other body fluids, suggesting that extracellular miRNAs are not only passively released outside the cells. Several studies have shown that, in fact, the miRNA content of extracellular vesicles does not simply reflect the miRNA repertoire of the cells of origin and that some miRNAs are selectively exported or retained within the cell (Guduric-Fuchs et al. 2012). However, the mechanism whereby specific miRNAs are selectively exported is still unknown. As a consequence, because several secreted miRNAs are undetectable in the cytoplasm of the secreting cells, it is almost impossible to identify specific miRNA signatures for a given tissue directly from the body fluids unless we have determined all exported miRNAs for each tissue (Cookson et al. 2012). In agreement, analysis of the human miRNA disease database has revealed that only a few of the miRNAs deregulated in blood are also reported as deregulated in solid tissues derived from individuals with the same diseases (Keller et al. 2011).

From the analysis of global miRNA expression signatures, it has been found that some circulating miRNA levels are proportional to the degree of severity of the pathology, such as drug-induced liver injury (Wang et al. 2009), cardiovascular infection (Van Aelst and Heymans 2013), diabetes (Guay and Regazzi 2013), cancer (Ferracin et al. 2010) and Alzheimer’s disease (Geekiyanage et al. 2012), or for prenatal diagnosis of preeclampsia (Gunel et al. 2011). However, the selection of biomarkers in nutrigenomics needs to reflect more subtle changes in homoeostasis representing the relationship between nutrition and health, or nutrition and disease, and until now, the most relevant body fluids for miRNA quantification in response to nutrition have not been clearly defined (Table 1).

**Table 1:** Studies mentioned in the review

References	Interventions	Circulating miRNAs	Biofluids	Associated parameters
Bye et al. (2013)	Physical activity	miR-210, miR-125a, miR-29a, let-7d, miR-21, miR-222, miR-652, miR-151	Blood	Low VO_2max_ level
Bye et al. (2013)	Acute exhaustive exercise and sustained aerobic exercise training	miR-146a, miR-20a	Blood	Peak exercise capacity and cardiorespiratory fitness
Dhahbi et al. (2013)	Calorie restriction (mouse)	hsa-miR-151a-3p, hsa-miR-151a-5p, hsa-miR-181a-1-3p, hsa-miR-3607	Blood	Calorie restriction
Ortega et al. (2013)	Surgery-induced weight loss	miR-140-5p, miR-122, miR-193a-5p, miR-16-1, miR-221 and miR-199a-3p	Blood	Weight loss
Milagro et al. (2013)	Weight loss	miR-935, miR-4772, miR-874, miR-199b, miR-766, miR-589 and miR-148b	Blood mononuclear cells	Weight loss
Singh et al. (2012)	Diet modulation (mouse)	rno-miR-351, mmu-miR-487b, mmu-miR-467a, mmu-miR-27b*, mmu-miR-148a, mmu-miR-145, mmu-miR-183, mmu-miR-133a, mmu-miR-133a-2, mmu-miR-150, mmu-miR-672, mmu-miR-181a-1*, rno-miR-664, mmu-miR-455, mmu-miR-138*, mmu-let-7 g*	Caecum	Microbiota composition
Tarallo et al. (2014)	Different dietary habits (vegans, vegetarians and omnivorous)	miR-92a	Stools or plasma	Dietary habits vegans > vegetarians > omnivorous
Tome-Carneiro et al. (2013)	Type 2 diabetic patients receiving one-year supplementation with resveratrol-containing grape extract	miR-21, miR-181b, miR-663, miR-30c2, miR-155 and miR-34a	Blood mononuclear cells	Down-regulation of pro-inflammatory cytokines in PBMC
Ryu et al. (2011)	Dietary zinc deprivation and repletion	miR-10b, miR-155, miR-200b, miR-296-5p, miR-375, miR-92a, miR-145, miR-204, and miR-211	Blood	Dietary zinc intake
Enquobahrie et al. (2011)	Pregnant women, categorised by low or high plasma calcitriol level	miR-589, miR-601, miR-573, miR-138, miR-320d, miR-196a*, miR-92b, miR-423-3p, miR-484, miR-93, miR-574-5p	Blood	Levels of plasma vitamin D
Beckett et al. (2015)	Vitamin D intake	let-7a/b	Blood	Correlated with vitamin D receptor polymorphisms

## Circulating miRNAs might be used in dietary or lifestyle intervention studies

Nutrigenomic studies aim at understanding how nutrition and lifestyle influence metabolic pathways and homoeostatic control and how this regulation fails in the early phases of diet-related disease. As a consequence, biomarkers in nutrigenomics must support evidence-based dietary intervention strategies for restoring health and fitness and for preventing diet-related diseases. Based on accumulating evidence, together with unhealthy diets, physical inactivity is key risk factors for the major noncommunicable diseases such as cardiovascular diseases, cancer and diabetes. Physical activity is difficult to quantify in human, and the use of blood miRNAs has been tested to classify healthy individuals according to their VO_2max_ level (peak oxygen consumption), a good indicator of sedentarity (Rogers et al. 1990). It was found that miR-210, miR-222 and miR-21 were increased in healthy subjects with low VO_2max_ level (Bye et al. 2013). Interestingly, miR-222 was significantly correlated with self-reported habitual exercise intensity. The lack of association between these miRs and other fitness variables and traditional cardiovascular risk factors (e.g. cholesterol, smoking habit, obesity) suggested that they may have potential as biomarkers of fitness level (i.e. sedentarity) and thus could be also associated with future cardiovascular diseases (Bye et al. 2013). In addition, analysis of signatures of circulating miRNAs in response to acute exhaustive exercise and sustained aerobic exercise training confirmed their potential use as biomarkers of exercise training and demonstrated that miR-146a and miR-20a are quantitatively correlated with peak exercise capacity and cardiorespiratory fitness. In line with these results, it was also found that experimental physical inactivity over 5 days in healthy humans (e.g. dry immersion protocol) is sufficient to detect disturbances in endothelial functions associated with a selective increase in blood endothelial extracellular vesicles (Navasiolava et al. 2010). As extracellular vesicles contain miRNAs (Jaiswal et al. 2012), they might participate in the blood miRNA signature associated with inactivity.

In Western societies, there has been a large increase in the proportion of middle- and old-age population over the last century and increase in age-related diseases, such as cancers, diabetes and Alzheimer’s disease. Caloric restriction, a decreased caloric intake without malnutrition, is the only environmental stimulus known to positively interfere with the ageing process (McCay et al. 1989). Several reports have demonstrated alterations in miRNA levels during mammalian ageing and senescence (Grillari and Grillari-Voglauer 2012) and have shown that circulating miRNAs could be involved in ageing (Li et al. 2011; Olivieri et al. 2012). In order to determine the effect of calorie restriction on age-associated circulating miRNAs, serum miRNA profiles were compared between young mice, old mice and old mice maintained on calorie restriction (Dhahbi et al. 2013). Circulating levels of 48 age-related miRNAs were reversed by calorie restriction. These miRNAs have been previously associated with cancer, neurodegenerative, cardiovascular and inflammatory disorders, which are all pathologies associated with ageing. Thus, age-related circulating miRNAs may participate in the development of age-induced diseases, and modulation of their level in the serum may underlie the anti-ageing effect of calorie restriction (Dhahbi et al. 2013).

In patients suffering from morbid obesity, diet-induced weight loss and gastric bypass-induced weight loss did not show the same effect on circulating miRNAs. Surgery-induced (but not diet-induced) weight loss led to a marked decrease in miR-140-5p, miR-122, miR-193a-5p and miR-16-1 and up-regulation of miR-221 and miR-199a-3p (Ortega et al. 2013). Since surgery-induced weight loss is known to improve most obesity-associated complications, whereas, in most cases, conventional diet-induced weight loss does not, it is suspected that these six miRNAs could predict outcomes in metabolic diseases. Recently, it has been found that miRNAs in peripheral blood mononuclear cells (PBMC) could be used as pronostic values for monitoring weight control (Milagro et al. 2013). Differential baseline expression of several miRNAs was found to explain the differences between responders and nonresponders to weight loss. Two miRNAs (miR-935 and miR-4772) were up-regulated in the nonresponder group, and three others were down-regulated (miR-223, miR-224 and miR-376b). The levels of circulating miR-935, miR-4772, miR-874, miR-199b, miR-766, miR-589 and miR-148b were correlated with weight loss.

Together with diet, it is now established evidence of a complex interplay between the gut microbiota and health, and that microbiota alterations are associated with disease states (e.g. obesity and diabetes) (Tilg and Kaser 2011). The balance of benefit and harm for the host depends on the overall state of the microbial community in terms of its distribution, diversity, species composition and metabolic outputs (Le Chatelier et al. 2013). Furthermore, it has become clear that diet can have a major influence on microbial community composition in both the short and long term, which should open up new possibilities for dietary health manipulation (Delzenne et al. 2013). In this context, the impact of endogenous microbiota on the global expression of caecal miRNAs in vivo has been evaluated by using germ-free and conventionally raised mice (Singh et al. 2012). Murine miRNA profiles in the caecum included several species and a subset of 16 miRNAs were differentially expressed between the two groups of mice. Many of their putative mRNA target encoded genes were involved in the regulation of the intestine barrier function (i.e. glycosylation enzymes, junctional proteins, immune response). This study provided the first proof-of-concept that caecal miRNAs could be relevant biomarkers to monitor the impact of diet modulation on the interaction between the intestine and the gut microbiota. It has been shown that caecal miRNAs are protected from RNase degradation in exosomes which can explain part of their stability in faeces (Koga et al. 2011). Recently, Tarallo et al. (2014) compared the expression of seven miRNAs in plasma and stool samples in a group of 24 healthy volunteers characterized by different dietary habits (vegans, vegetarians and omnivorous), all groups with similar age and sex distribution (Tarallo et al. 2014). miR-92a was differentially expressed in both plasma and stool samples and was more highly expressed in vegans and vegetarians compared to omnivores. More remarkable, miR-92a expression was strongly inversely related to cheese consumption (including dairy products) in both stool and sample plasma. This miRNA was also associated with lower BMI. In the same study, miR-16, miR-21, miR-34a and miR-222 were associated with dietary (e.g. meat/fish consumption, weekly intake of vegetables, fruit intake) and lifestyle factors, but not consistently in both stool and plasma. This study established that miRNA modulation by specific diet may be detected in stool samples.

## Diet supplementation and circulating miRNAs

The impact of diet supplementation on miRNA expression has been extensively studied at the cellular or tissue level, and specific miRNA signatures have been found (Garcia-Segura et al. 2013). In the peripheral blood, few studies have evaluated whether miRNAs could be used to monitor the beneficial effect of specific supplementations. It was found that in type 2 diabetic patients receiving one-year supplementation with resveratrol-containing grape extract (GE-RES), the observed down-regulation of pro-inflammatory cytokines in PBMC was concomitant with higher levels of miRNAs involved in the regulation of the inflammatory response (i.e. miR-21, miR-181b, miR-663, miR-30c2, miR-155 and miR-34a) (Tome-Carneiro et al. 2013). The possibility of following blood cell miRs that influence inflammatory pathways by dietary supplementations adds new perspectives for the treatment of inflammatory-related diseases. In this context, it was also demonstrated that dietary zinc intake influences miRNAs circulating in serum in human (Ryu et al. 2011). Nine miRNAs (miR-10b, miR-155, miR-200b, miR-296-5p, miR-375, miR-92a, miR-145, miR-204, and miR-211) were found to be regulated by dietary zinc deprivation and repletion in opposite modes (Ryu et al. 2011). However, the studies investigating the correlation between serum levels of vitamin D and miRNA expression in humans have given contrasted results. Indeed, in a study of 13 pregnant women, categorised by low (≤25.5 ng/ml) or high (≥31.7 ng/ml) plasma calcitriol level, 11 miRNAs were differentially regulated (Enquobahrie et al. 2011). Conversely, Jorde et al. (2012) were unable to demonstrate a consistent effect of high doses of vitamin D3 (cholecalciferol) supplementation for 12 months on the expression profile of miRNAs in plasma of 10 subjects (Jorde et al. 2012). The authors postulated that because only 12 miRNAs had been selected for the analyses, it was in fact difficult to conclude a global absence of plasma miRNA regulations in their study (Jorde et al. 2012). Recently, Beckett et al. (2015) made the hypothesis that circulating levels of the miRNAs could be correlated with polymorphisms of two common vitamin D receptors (VDR) (Beckett et al. 2015). They demonstrated that the negative correlation between vitamin D intake and let-7a/b expression in a cohort of 200 subjects was associated with the VDR genotype. This study highlights the importance of considering underlying genotypic variance in miRNA expression studies.

## Overview of main preanalytical considerations that are important to take into account before miRNA screening

As there is presently very few studies on circulating miRNAs in response to dietary substances (Table 1), it is difficult to firmly conclude that miRNAs might be used as biomarkers in nutritional studies because these studies have not yet been reproduced and compared. However, based on substantial number of studies that have demonstrated that the level of blood miRNAs is affected in a wide range of disorders, it is now clear that some blood preanalytical steps are important parameters that affect miRNA detection and quantification (Zhao et al. 2014). First, beside important basic technical considerations (e.g. serum *vs* plasma (Wang et al. 2012), hemolysis of plasma and serum samples (Kirschner et al. 2011, 2013a), kits used for RNA isolation (Moret et al. 2013) and quantification, methods for miRNA screening (microarray, low-density array, sequencing) (Leidner et al. 2013), technologies for validation by qRT-PCR (Roberts et al. 2014) and data normalisation (Kirschner et al. 2013b), it is now clear that two different populations of miRNAs coexist in biofluids (i.e. packed inside vesicles *vs* vesicle free) that might have specific signatures (Fig. 1). By using differential centrifugation and size-exclusion chromatography to systematically characterize circulating miRNA complexes in human plasma and serum, Arroyo (2011) found that vesicle-associated plasma miRNAs represent the minority, whereas potentially up to 90 % of miRNAs in the circulation are present in a nonmembrane-bound form (Arroyo et al. 2011). This result was further confirmed in (Turchinovich et al. 2011).

Until now, the majority of the studies have focused on the whole miRNA signature. However, it has recently been demonstrated that cells selectively export a subset of miRNAs in vesicles (Guduric-Fuchs et al. 2012) and thus the global analysis of circulating miRNAs might result in a complex signature that superimposed different miRNAs variations according to their mode of secretion. This could contribute to large variances in miRNA abundances between individuals (Ashby et al. 2014). Second, it has been demonstrated that miRNAs might have sex-specific associations (Wang et al. 2013; Tarallo et al. 2014) and thus that considering both men and women in the same studies for miRNA screening might introduce discrepancy among the results. In addition, it has also become obvious from studies analysing circulating miRNAs in the context of metabolic diseases that relevant miRNAs might be associated with the demographic characteristics of the subjects (Prabu et al. 2015; Zhu and Leung 2015); for example, Caucasian and Asian populations have unique metabolic profiles, these latter being at elevated risk of diabetes and having specific nutritional habits (Gujral et al. 2013). Taken these considerations altogether, it appear that there is still a long route from proof-of-principle to the identification of reliable miRNAs for monitoring the impact of specific diet on the metabolism. Finally, data from the literature indicated that the same circulating miRNAs, found to be responsive to dietary modulations, are also found altered in different pathological contexts, suggesting that they are not specific to a given disease. This result can be explain by the fact that not all miRNAs are stable in the blood and thus measurable (Koberle et al. 2013) and that only a handful of them is exported from cells (Guduric-Fuchs et al. 2012). In agreement, Blondal et al. (2013) identified only 114 miRNAs consistently expressed in 1500 serum and plasma samples (Blondal et al. 2013), although more than 1000 miRNAs are recorded in the miRBase (mirbase.org). Thus, altered miRNA level in the blood may be more related to a general alteration of the metabolism and to systemic inflammation that affect common organs in various pathological states (cardiovascular disease, cancer, diabetes, ageing, obesity, etc.) than to a specific disease. Therefore, the identification of nutrients and dietary or lifestyle interventions that are able to reverse the level of altered circulating miRNAs would indicate that the interventions have a positive impact on the metabolism and might permit to identify dietary factors able to treat or reduce the progression of the disease.

## Conclusions

The use of noninvasive indicators of nutritional status, including dietary and lifestyle interventions, is urgently needed as a large branch of nutrition is based on observational studies. Recently, it was discovered that part of the noncoding genome (including miRNAs) is exported outside the cells and is stable enough to be amplified and sequenced and, in some circumstances, to be re-incorporated into distant cells where an array of biological processes can be affected. Since this discovery, a growing number of studies have demonstrated that circulating miRNA levels are good biomarkers for cancers, neurological disorders and metabolic diseases. Although there is still a number of technical issues associated with miRNA profiling in biofluids (Pritchard et al. 2012; Cheng et al. 2013), their use as indicators in nutritional studies needs to be urgently evaluated as a number of relevant studies indicate that miRNAs might be promising biomarkers to monitor the impact of lifestyle or dietary interventions. In addition, because a significant number of studies have already identified altered circulating miRNAs associated with specific diseases, it is tempting to postulate that the identifications of nutrients that can reverse these alterations might participate in the treatment of these diseases or alter disease progression.
